# Plasticity of the Cuticular Transpiration Barrier in Response to Water Shortage and Resupply in *Camellia sinensis*: A Role of Cuticular Waxes

**DOI:** 10.3389/fpls.2020.600069

**Published:** 2021-01-11

**Authors:** Yi Zhang, Zhenghua Du, Yanting Han, Xiaobing Chen, Xiangrui Kong, Weijiang Sun, Changsong Chen, Mingjie Chen

**Affiliations:** ^1^Henan Key Laboratory of Tea Plant Biology, College of Life Sciences, Xinyang Normal University, Xinyang, China; ^2^Horticultural Plant Biology and Metabolomics Center, Haixia Institute of Science and Technology, Fujian Agriculture and Forestry University, Fuzhou, China; ^3^Tea Research Institute, Fujian Academy of Agricultural Sciences, Fuan, China; ^4^Anxi College of Tea Science, Fujian Agriculture and Forestry University, Fuzhou, China

**Keywords:** *Camellia sinensis*, cuticular transpiration rate, cuticle, drought, epicuticular waxes, intracuticular waxes, rehydration, wax coverage

## Abstract

The cuticle is regarded as a non-living tissue; it remains unknown whether the cuticle could be reversibly modified and what are the potential mechanisms. In this study, three tea germplasms (*Wuniuzao*, *0202-10*, and *0306A*) were subjected to water deprivation followed by rehydration. The epicuticular waxes and intracuticular waxes from both leaf surfaces were quantified from the mature 5th leaf. Cuticular transpiration rates were then measured from leaf drying curves, and the correlations between cuticular transpiration rates and cuticular wax coverage were analyzed. We found that the cuticular transpiration barriers were reinforced by drought and reversed by rehydration treatment; the initial weak cuticular transpiration barriers were preferentially reinforced by drought stress, while the original major cuticular transpiration barriers were either strengthened or unaltered. Correlation analysis suggests that cuticle modifications could be realized by selective deposition of specific wax compounds into individual cuticular compartments through multiple mechanisms, including *in vivo* wax synthesis or transport, dynamic phase separation between epicuticular waxes and the intracuticular waxes, *in vitro* polymerization, and retro transportation into epidermal cell wall or protoplast for further transformation. Our data suggest that modifications of a limited set of specific wax components from individual cuticular compartments are sufficient to alter cuticular transpiration barrier properties.

## Introduction

The plant cuticle is a lipophilic layer coating essentially all aerial organs and acts as an interface between plants and environment. Besides protection from UV radiation, insects, pathogens, and environmental contaminants (Eigenbrode and Espelie, 1995; Krauss et al., 1997; Neinhuis and Barthlott, 1997; Müller, 2006; Zhang et al., 2019; Lino et al., 2020), the cuticle serves as a primary barrier to restrict non-stomatal water loss and facilitate plants to survive through drought stress (Riederer and Schreider, 2001; Nawrath et al., 2013; Chen et al., 2020). The cuticle is a composite material chiefly made of cutin, waxes (epicuticular and intracuticular waxes), and polysaccharides (Guzmán et al., 2014a, b; Fernández et al., 2017). Cutin is a polyester cross-linked by ester bonds of C16 and C18 hydroxy-fatty acids (Pollard et al., 2008; Fich et al., 2016); polysaccharides are found in the entire cuticle of leaves from different species (Guzmán et al., 2014a, b; Philippe et al., 2020). The outer cuticle surface is coated with a layer of epicuticular waxes (EWs) while intracuticular waxes (IWs) are embedded into the cuticle (Kulkarni et al., 2018). Unlike EWs, IWs are in the cuticle interior and thus cannot be stripped off by adhesives such as gum arabic, collodion, or cellulose acetate (Buschhaus and Jetter, 2011). Cuticular waxes generally include aliphatic compounds (fatty acids, primary alcohols, alkanes, aldehydes, and ketones) and some alicyclic compounds (triterpenoids and sterols) (Samuels et al., 2008), and play major roles to form cuticular transpiration barrier, while cutin polymer contributes minorly (Riederer and Schreiber, 1995; Richardson et al., 2007). The cuticular wax composition is expected to affect cuticular transpiration barrier properties. Earlier studies used bulk waxes under the assumption that they could represent the surface; possible differences between EWs and IWs were ignored (Jetter and Schäffer, 2001; Buschhaus and Jetter, 2012; Jetter and Riederer, 2016; Hasanuzzaman et al., 2017; Bueno et al., 2019; Romero and Rose, 2019). As a result, it remains controversial how wax composition is correlated with cuticular transpiration barrier (Riederer and Schreider, 2001; Sánchez et al., 2001; Kosma et al., 2009; Seo et al., 2011; Jetter and Riederer, 2016; Hasanuzzaman et al., 2017).

The establishment of an efficient isolation method to differentiate EWs from IWs necessitates a differential analysis of the cuticular transpiration barrier and its relationship with cuticular composition. These studies demonstrated that the adaxial IWs and abaxial EWs constitute major cuticular transpiration barrier, while the adaxial EWs and abaxial IWs are not major contributors to leaf transpiration barrier (Vogg et al., 2004; Buschhaus et al., 2015; Jetter and Riederer, 2016; Zhang et al., 2020). Aliphatic compounds from adaxial IWs are correlated mainly with cuticular transpiration barrier (Jetter and Riederer, 2016), while the alicyclic compounds did not contribute to cuticular transpiration barrier (Buschhaus and Jetter, 2012). Consistent with this conclusion, the permeation of active ingredients is limited by very long-chain aliphatic compounds rather than alicyclic wax compounds (Staiger et al., 2019). Recently, we demonstrated that abaxial cuticle showed different transpiration barrier organization from its adaxial counterpart: the abaxial EWs is another major transpiration barrier while the abaxial IWs contribute minorly (Zhang et al., 2020). Adaxial IWs showed higher coverage in very long chain fatty acids (VLCFAs), 1-alkanol esters, and glycols than that of abaxial IWs, suggesting that these waxes could contribute to their differential barrier properties under normal growth conditions (Zhang et al., 2020).

Cuticular wax composition can be modified by developmental programs or environmental cues (Weissflog et al., 2010; Buschhaus et al., 2015; Zhu et al., 2018; Bourgault et al., 2020; Chen et al., 2020). In response to drought stress, plants undergo diverse wax modifications for transpiration barrier reinforcement (Aharoni et al., 2004; Zhang et al., 2005; Bueno et al., 2019; Li et al., 2019). It is generally assumed that different germplasms within the same plant species could show similar patterns for cuticular wax modification in response to drought stress. However, crucial aspects of cuticle modification, especially the mechanisms to regulate wax deposition at individual cuticular compartments, remain largely unknown. Recently, tea tree (*Camellia sinensis*) emerged as a good system to study plant cuticle (Zhu et al., 2018; Chen et al., 2020). In this study, three germplasms of *Camellia sinensis* (*Wuniuzao*, *0202-10*, and *0306A*) were subjected to water deprivation (WD), then irrigation was resumed for plant recovery. Cuticular transpiration rates and wax compositions were quantified simultaneously, and correlation analyses were conducted. We found that the cuticular transpiration barriers were reversibly modified by drought and rehydration treatment; in response to drought stress, the initial weak cuticular transpiration barriers were preferentially modified, while the original major cuticular transpiration barriers were either reinforced or remained unaltered. This work offers new insight about dynamic cuticle modification and the potential underlying mechanisms in response to water status changes.

## Materials and Methods

### Plant Materials

Initially, we developed an *in vitro* method to measure water loss from tea flush (one bud and two leaves), then applied to screen more than 100 tea germplasms. *Camellia sinensis* germplasm *Wuniuzao*, *0306A*, and *0202-10* showed different levels of water loss and were selected in this study for further characterization. They were clonal propagated in the tea garden at the Tea Research Institute of Fujian Academy of Agricultural Sciences (Fu’an, China; 119.3°E, 27.1°N). One-year-old seedlings were transplanted into plastic pots on March 2018, with three seedlings per pot. The pot size was 25 cm in diameter and 25 cm in height and filled with 5 kg of local red soil. The pots were half-buried into the tea garden and grown additional 7 months for full root establishment.

### Drought and Rehydration Treatment

Tea pots were moved from garden into a glasshouse on October 2018, fully soaked, then randomly divided into well-watered control (WW) and water-deprivation (WD) groups. The WW plants were irrigated daily with 500 ml water per pot, while the water was withheld to initiate WD treatment. Daily temperature and humidity inside the glasshouse showed considerable fluctuations (Supplementary Figures 1A,B). The pot weights from WD groups were recorded daily, and the absolute soil water contents were calculated (Supplementary Figure 2A). After 12 days of WD treatment, water was resumed to terminate drought treatment.

### Cuticular Transpiration Rate Measurement

The cuticular transpiration rates from the detached 5th leaf were measured from WW control, day 8 of WD (D8), and day 10 of rehydration (RE) as the methods of Zhang et al. (2020). Briefly, shoots with one bud and seven leaves were removed, and the cutting end of the shoots were immersed in water and equilibrated overnight. The next day, abaxial leaf surfaces were evenly sprayed with 50 μM ABA, and after leaving for 1 h, excess water was gently blotted dry by a soft tissue. The 5th leaf was detached from each shoot, and leaf stem excision site was immediately sealed with Vaseline. Each leaf was photographed for the measurement of leaf area (A) by ImageJ software. The initial water-saturated fresh weight (W_i_) was recorded, leaves were then placed in a dark room (25°C, 45% humidity), hourly leaf weight was determined gravimetrically, and the experiment lasted for 10 h (W_t 0_,_1_,_… 10_). Each treatment group included four to six leaves. Before ending the experiment, the leaves were dried at 80°C for 24 h to obtain individual leaf dry weight (W_d_). The relative leaf water content was calculated by the formula RWC = [(W_t_ − W_d_)/(W_i_ − W_d_)] × 100%. Leaf relative water deficit (RWD) was calculated by the formula RWD = [1 − RWC] × 100% (Burghardt and Riederer, 2003).

### Epicuticular and Intracuticular Wax Isolation

Epicuticular waxes and IWs were isolated from the 5th leaf of WW control, day 8 of WD, and day 10 of RE as Zhang et al. (2020). Delipidated gum arabic was dissolved in water to a final concentration of 90% (w/v), and aqueous solution was evenly applied to leaf surface to form a film. Dry polymer film was peeled off and collected, then extracted in 21 ml of chloroform/water (2:1, v/v) containing 75 μg of internal standard *n*-tetracosane (Sigma-Aldrich, St. Louis, MO, United States). After vigorous agitation and phase separation, the organic phase was collected into a new glass tube. The residue was extracted one more time in 4.5 ml extraction buffer; the organic phase was combined and evaporated in a rotary evaporator (Labconco, Kansas, MO, United States) to obtain EWs. The adaxial EW was removed first, followed by abaxial EW.

After EW removal from both surfaces, IWs were extracted with chloroform rinsing. The adaxial IWs and the abaxial IWs were subsequently extracted. The collected chloroform solution was evaporated dry to obtain adaxial and abaxial IWs.

### Wax Analysis

Before GC–MS and GC–FID analysis, wax samples were derivatized by N,O-bis(trimethylsilyl)-trifluoroacetamide (BSTFA, Aldrich, GC grade) plus 1% trimethylchlorosilane (Aldrich) in pyridine (Aldrich, 99.8%, anhydrous). Individual wax was identified from MS data by comparing mass spectra with the NIST 14 database. Individual wax was quantified from FID data by normalizing the peak area with that of internal standard. DB-1 column (30 m × 0.25 mm × 0.25 μm; Agilent, CA, United States) was used for GC–MS and GC–FID analysis. The helium was used as carrier gas with flow rates of 1.2 and 1.7 ml min^–1^ for GC–MS and GC–FID, respectively. The flow rates of hydrogen, nitrogen, and zero air were 40, 30, and 400 ml min^–1^, respectively. The initial oven temperature was set at 70°C, raised at 10°C min^–1^ to 200°C, held for 2 min, then raised at 3°C min^–1^ to 320°C, held for 20 min, then returned to 70°C for next sample injection. The MS detector setting was EI, 70 eV; and ionization source temperature, 230°C.

### Statistical Analysis

ANOVA within Excel software was used to calculate mean and SE. Significance was determined by one-way ANOVA based on Duncan’s multiple range tests. Regression analysis between cuticular transpiration rate and wax composition was performed by SPSS (V17.0; SPSS, IBM, Armonk, NY, United States). Bivariate correlations based on Pearson’s correlation (two-tailed) were used to determine the significance of correlations among different variables.

## Results

### The Phenotypic Changes During Water Deprivation and Rehydration

Before initiation of water deprivation treatment, tea pot soil was fully soaked, and the absolute soil water content (ASW) reached 44%. After initiation of WD, ASW decreased rapidly, and reached 34.1% at day 5 (D5) of WD (Supplementary Figure 2A). All these three tea germplasms started to show variable drought symptoms during daytime and recovered after sunset (Supplementary Figure 2C). At day 8 (D8) of WD, ASW reached 32% (Supplementary Figure 2A) and leaf relative water contents (RWCs) ranged from 48 to 57% (Supplementary Figure 2B). At day 12 (D12) of WD, ASW dropped to 28% (Supplementary Figure 2A), and leaf RWCs were in the range of 39 to 44%. Leaves became chlorotic, and the lower part of leaf started to become senescent (Supplementary Figure 2C). At this point, the water was resumed. Ten days later, *Wuniuzao* recovered and its leaf RWC reached 85%, a level similar to that before WD treatment. Both *0202-10* and *0306A* partially recovered (Supplementary Figure 2B).

### The Coverages of Epicuticular Waxes and Intracuticular Waxes Were Differentially Altered in Response to Water Deprivation and Rehydration Treatment

**FIGURE 1:**
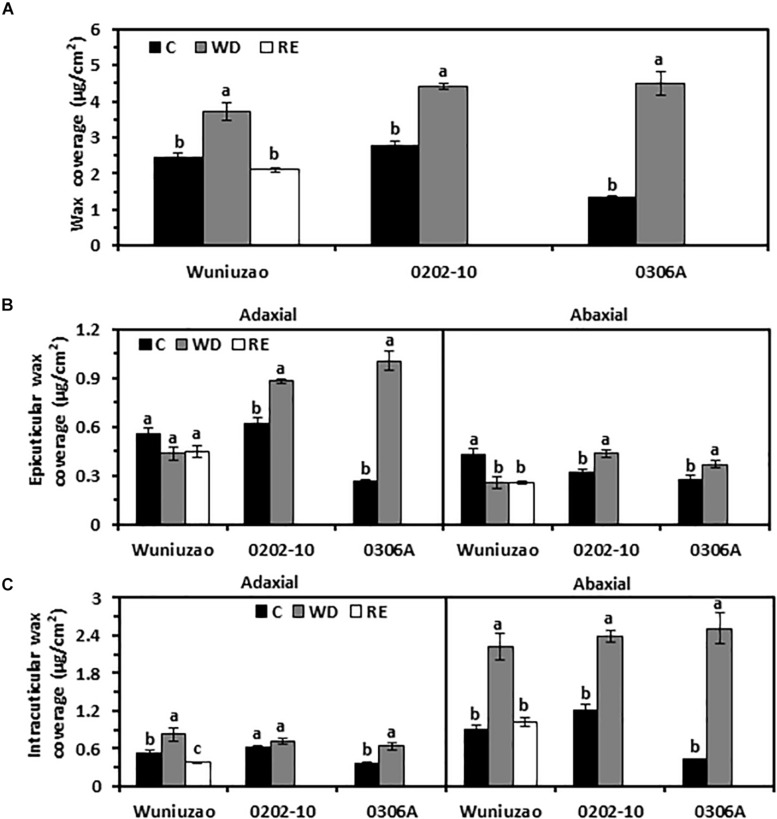
Cuticular wax coverage changes from *Wuniuzao*, *0202-10*, and *0306A* under well-watered control (C), water deprivation (WD), and rehydration (RE). **(A)** Total cuticular wax coverage changes from three tea germplasms under C, WD, and RE. **(B)** Epicuticular wax coverage from three tea germplasms under C, WD, and RE. **(C)** Intracuticular waxes coverage from three tea germplasms under C, WD, and RE. Data are expressed as mean ± SE (*n* = 4). Different letters within the same germplasm indicate statistical significance (*P* < 0.05).

At D8 of WD treatment, the total wax coverages from these three tea germplasms were significantly increased compared with WW control (Figure 1A and Supplementary Table 1). Under WW condition, *0306A* showed the lowest wax coverage; at D8 of WD, its total wax coverage increased 3.4-fold. Meanwhile, the total wax coverage from *Wuniuzao* and *0202-10* only increased 53% and 60%, respectively (Figure 1A). As a result, at D8 of WD, the three germplasms showed similar levels of total wax coverage.

Each cuticular compartment showed different trends of wax accumulation. After WD treatment, the EW coverage from *0202-10* and *0306A* increased significantly from both surfaces compared with their respective WW control; in contrast, the adaxial EW coverage of *Wuniuzao* remained unaltered while its abaxial EW coverage was significantly reduced compared with WW control (Figure 1B and Supplementary Table 1). The IW coverages from all three germplasms were significantly increased by WD treatment except the adaxial IW of *0202-10* (Figure 1C). The abaxial IW coverages showed much greater increase compared with respective adaxial surface (Figure 1B).

After RE treatment, the wax changes in *Wuniuzao* were also quantified. After 10 days of RE treatment, total wax coverage reached a similar level as WW control and significantly lower than that of WD (Figure 1A). RE treatment did not affect the EW coverages from both leaf surfaces; in contrast, the IW coverages from both leaf surfaces were significantly reduced compared with WD-treated plants (Figures 1A–C).

**FIGURE 2:**
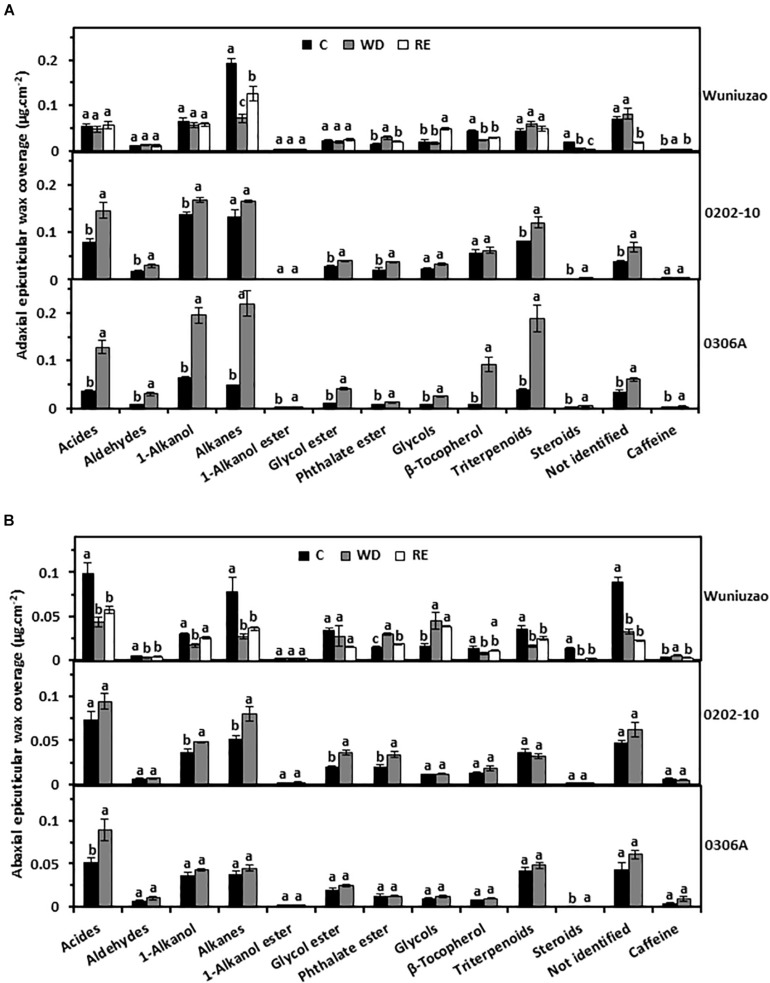
The epicuticular wax coverages of *Wuniuzao*, *0202-10*, and *0306A* under well-watered control (C), water deprivation (WD), and rehydration (RE). **(A)** The adaxial epicuticular wax coverage. **(B)** The abaxial epicuticular wax coverage. Data are expressed as mean ± SE (*n* = 4). Different letters within the same germplasm indicate statistical significance (*P* < 0.05).

Next, the wax chemical classes were examined. The tea leaf cuticular waxes include acids, aldehydes, 1-alkanols, alkanes, 1-alkanol esters, glycol esters, phthalate esters, glycols, β-tocopherol, triterpenoids, steroids, caffeine, and some unknown components (Kulkarni et al., 2018; Zhu et al., 2018; Zhang et al., 2020). The majority of the chemical classes from adaxial EWs of *0202-10* and *0306A* were significantly increased by WD treatment (Figure 2A, middle and lower panels); in contrast, most of these chemical classes from *Wuniuzao* were not affected by WD treatment – some waxes were even reduced by WD treatment (Figure 2A, upper panel).

The abaxial EW coverage increased from *0202-10* and *0306A* after WD, but was ascribed to different chemical classes (Figure 2B, upper panel right side and Supplementary Table 1): 1-alkanol, alkane, glycol ester, and phthalate ester were the major contributors in *0202-10* (Figure 2B, middle panel); in contrast, acids, and steroids became the major contributors in *0306A* (Figure 2B, lower panel). In *Wuniuzao*, eight out of 13 chemical classes were reduced, while the other five chemical classes were either unaltered or only slightly increased by WD treatment (Figure 2B, top panel).

**FIGURE 3:**
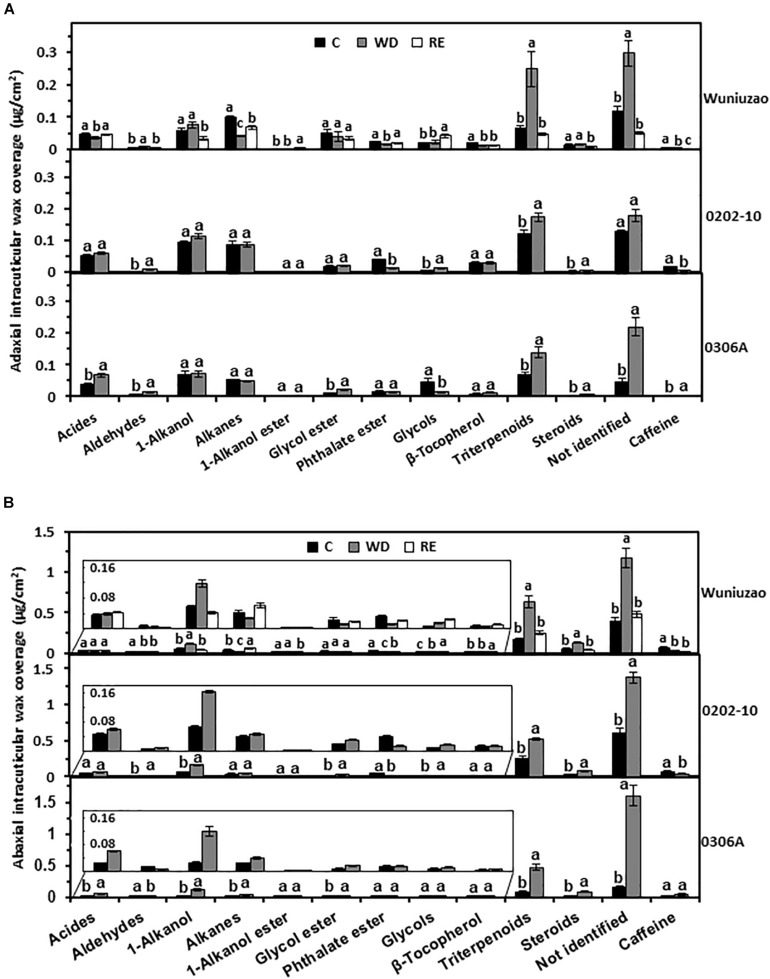
The intracuticular waxes coverage of *Wuniuzao*, *0202-10*, and *0306A* under well-watered control (C), water deprivation (WD), and rehydration (RE). **(A)** The adaxial intracuticular wax coverage. **(B)** The abaxial intracuticular wax coverage. Data are expressed as mean ± SE (*n* = 4). Different letters within the same germplasm indicate statistical significance (*P* < 0.05).

Triterpenoids from adaxial and abaxial IWs were commonly increased across these three germplasms after WD treatment (Figures 3A,B). In addition, the coverage of 1-alkanols and steroids from abaxial IWs was all increased by WD treatment (Figure 3B).

The RE treatment significantly reduced the adaxial and abaxial IWs of *Wuniuzao* (Figure 1B, lower panel, left side). Alkanes, phthalate esters, and triterpenoids showed reversible change from both surfaces; alkanes and phthalate ester were significantly decreased by WD treatment compared with WW control, and significantly increased by RE compared with WD treatment. In contrast, triterpenoids showed an opposite trend as alkanes and phthalate ester (Figures 3A,B, top panel and Supplementary Table 1).

Besides these common changes, some wax classes showed reversible changes only from adaxial IWs or abaxial IWs during the WD–RE cycle: in adaxial IWs, acids were markedly reduced by WD treatment, then increased by RE treatment; in contrast, aldehydes were noticeably increased by WD treatment, then decreased by RE treatment. In abaxial IWs, 1-alkanols showed similar trends as aldehydes from adaxial IW (Figures 3A,B, top panel).

Although the adaxial and abaxial EW coverage of *Wuniuzao* showed different changing trends during the WD–RE cycle (Figure 1B, top panel and Supplementary Table 1), phthalate ester and caffeine still showed reversible changes, which were increased by WD and decreased by RE (Figures 2A,B, upper panel). In contrast, alkanes from adaxial EWs and 1-alkanols from abaxial EW were decreased by WD and increased by RE (Figures 2A,B, upper panel).

### Cuticular Transpiration Rates Were Reversibly Altered by Water Deprivation and Rehydration Treatment

**FIGURE 4:**
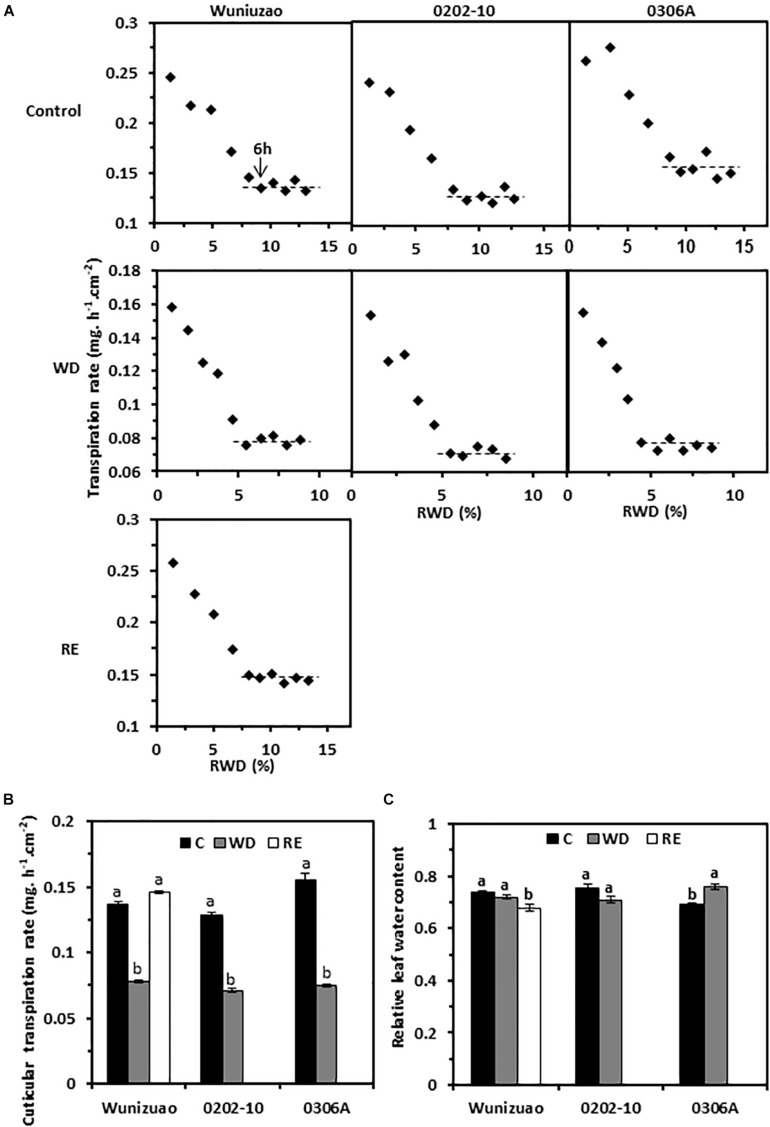
Cuticular transpiration rates from *Wuniuzao*, *0202-10*, and *0306A* under well-watered control (C), water deprivation (WD), and rehydration (RE). **(A)** Leaf drying curves of *Wuniuzao*, *0202-10*, and *0306A* from C, WD, and RE. The transpiration rate was plotted versus the relative water deficit (RWD). **(B)** Cuticular transpiration rates of *Wuniuzao*, *0202-10*, and *0306A* under different growth conditions. **(C)** Relative leaf water contents of *Wuniuzao*, *0202-10*, and *0306A* under different growth conditions. Data are expressed as mean ± SE (*n* = 6). Different letters within the same germplasm indicate as statistical significance (*P* < 0.05).

Transpiration rates from the detached 5th leaf were measured from WW control, D8 of WD, and D10 of RE, respectively; the leaf drying curve was monitored for 10 h post-excision. Despite dark and ABA pretreatments to promote stomata closure, stomata from water-equilibrated leaves remained partially open at the initial stage of post-excision and closed progressively after leaf excision. The total leaf transpiration rate decreased accordingly before stabilizing to a constant level. At the turning point of the leaf drying curve, the stomata reach maximum closure, and the residual stomata transpiration is negligible (Burghardt and Riederer, 2003; Zhang et al., 2020). Thus, the minimal leaf transpiration rate can be used as proxy of cuticular transpiration rate. In this study, the leaf transpiration rates reached a constant and minimum value after 6 h post-excision (Figure 4A). The average minimal leaf transpiration rates during the 6th to 10th h post-excision were used as proxy of total leaf cuticular transpiration rates. For WW control plants, the cuticular transpiration rates of *Wuniuzao*, *0202-10*, and *0306A* were 0.137 ± 0.002, 0.128 ± 0.003, and 0.155 ± 0.005 mg h^–1^ cm^–2^, respectively (Figures 4A, upper panel, B). At D8 of WD, their cuticular transpiration rates were decreased to 0.078 ± 0.001, 0.071 ± 0.001, and 0.075 ± 0.001 mg h^–1^ cm^–2^, respectively (Figures 4A, middle panel, B). At D10 of RE, the cuticular transpiration rate of *Wuniuzao* reached 0.146 ± 0.001 mg h^–1^ cm^–2^, a level similar to its WW control (Figures 4A,B, lower panel). Thus, cuticular transpiration barriers were increased by drought and reversed by RE. To examine whether changes in cuticular transpiration rates were due to the differences in leaf water contents, the relative leaf water content, expressed as the percentage of the net water content relative to saturated leaf weight, was calculated. We found that the relative leaf water contents were not significantly affected by WD except *0306A*, which showed a small but significant increase; the relative leaf water contents from *Wuniuzao* were slightly decreased by RE (Figure 4C). Thus, the changes of the cuticular transpiration rates from these three germplasms were not correlated with the changes in their relative leaf water contents.

On the leaf drying curves, the intersect between the initial steep declining phase and the later plateau phase represents the onset of maximum stomatal closure; the corresponding RWDs represent the minimal leaf water deficit that is required for maximal stomatal closure (RWD_SC_) (Figure 4A). Under WW control condition, the RWD_SC_ was in the range of 8.7–9.8%; after WD treatment, their values were reduced almost by half (4.8–5.7%). Thus, WD treatment sensitizes plants to water deficiency, and stomata were maximally closed at lower levels of water deficiency. After RE treatment, *Wuniuzao* reached maximal stomata closure at RWD of 8.3%, a level similar to its WW control.

### The Contributions of EWs to the Cuticular Transpiration Barriers Were Altered by Water Deprivation and Rehydration Treatments

**FIGURE 5:**
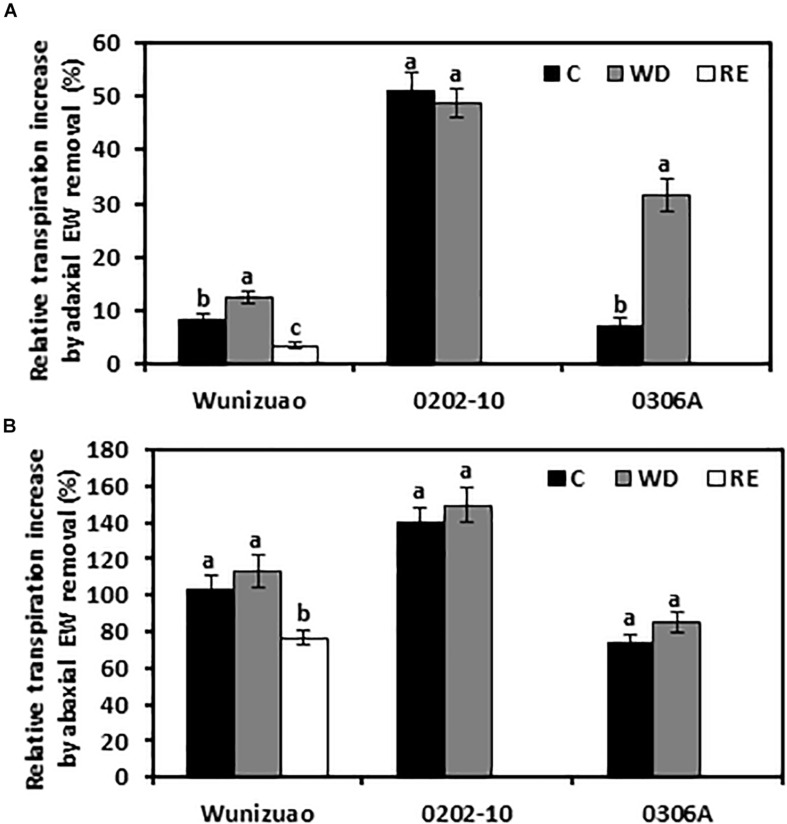
Relative cuticular transpiration rate increase after epicuticular waxes were removed. **(A)** Relative cuticular transpiration rate increase after removing adaxial epicuticular waxes. **(B)** Relative cuticular transpiration rate increase after removing abaxial epicuticular waxes. Data are expressed as mean ± SE (*n* = 6). Different letters within the same germplasm indicate statistical significance (*P* < 0.05). C, control; WD, water deprivation treatment; RE, rehydration treatment.

For the species analyzed, it has been demonstrated that adaxial IWs constitute the major transpiration barrier while adaxial EWs are not (Vogg et al., 2004; Buschhaus et al., 2015; Fernandez-Moreno et al., 2016; Jetter and Riederer, 2016; Zhang et al., 2020). Recently, we demonstrated that abaxial EWs constitute another major transpiration barrier while abaxial IWs are not (Zhang et al., 2020). To investigate whether the transpiration barrier properties of the EWs were affected by WD or RE treatments, the adaxial and abaxial EWs were individually stripped off by gum arabic, then the cuticular transpiration rates were measured. Because the cuticular transpiration rates from EWs cannot be directly measured by this method, the relative increase in EW transpiration, expressed as the ratio of net transpiration rate increase after EW removal relative to that of the intact leaf, was calculated. This ratio reflects the relative contribution of EWs to the total leaf cuticle transpiration barrier. After WD treatment, if the relative increase in EW transpiration were significantly higher than that of WW control, it can be inferred that the barrier properties of EWs were enhanced by the WD treatment. Under WW control conditions, when the adaxial EWs were removed by gum arabic, the relative increase of EW transpiration from *0202-10*, *Wuniuzao*, and *0306A* were 51 ± 3.1%, 8.2 ± 1.3%, and 7.1 ± 1.4%, respectively (Figure 5A). These data indicate that unlike *Wuniuzao*, *0306A*, or *Fuyun 6* (Zhang et al., 2020), adaxial EWs of *0202-10* constituted the major leaf transpiration barrier. After WD treatment, the relative increases of adaxial EW transpiration from *Wuniuzao* and *0306A* were significantly higher than that of WW control, indicating that WD treatment significantly improved the barrier properties of their adaxial EWs (Figure 5A). In contrast, the relative increases of adaxial EW transpiration from *0202-10* was not significantly different from that of WW control, indicating that its adaxial EW barrier property was unaffected by WD (Figure 5A).

The barrier properties of the abaxial EWs were also evaluated in the same manner. Under WW control conditions, the relative increases of abaxial EW transpiration from these three germplasms were all more than 73%, indicating that abaxial EWs constituted the major leaf transpiration barrier. Compared with WW control, the relative increases of abaxial EWs transpiration from these three tea germplasms were not significantly affected by WD treatment, suggesting that the transpiration barrier properties of abaxial EWs were not affected by WD treatment (Figure 5B).

The effects of RE treatment on the EW barrier properties were also evaluated for *Wuniuzao*. The relative increase of adaxial EWs transpiration was significantly lower compared with that of WW control or WD-treated plants (Figure 5A). A similar significant reduction was also observed after abaxial EW removal (Figure 5B). Thus, we concluded that RE treatment significantly reduced the transpiration barrier of both adaxial EWs and abaxial EWs in *Wuniuzao*.

### Principal Component Analysis

**FIGURE 6:**
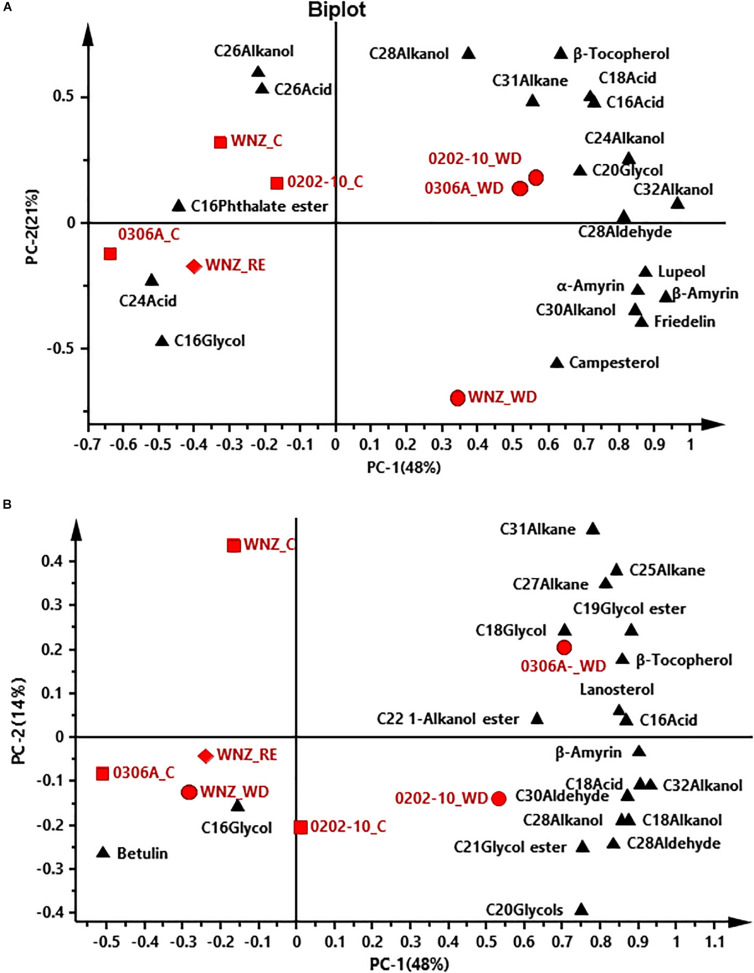
Principal component analysis (PCA) of total cuticular waxes **(A)** and adaxial epicuticular waxes **(B)**. WNZ, *Wuniuzao*; C, control, represented by solid square; WD, water deprivation treatment, represented by solid circle; RE, rehydration treatment, represented by solid diamond. The solid triangle represents individual wax position on PCA plot.

**FIGURE 7:**
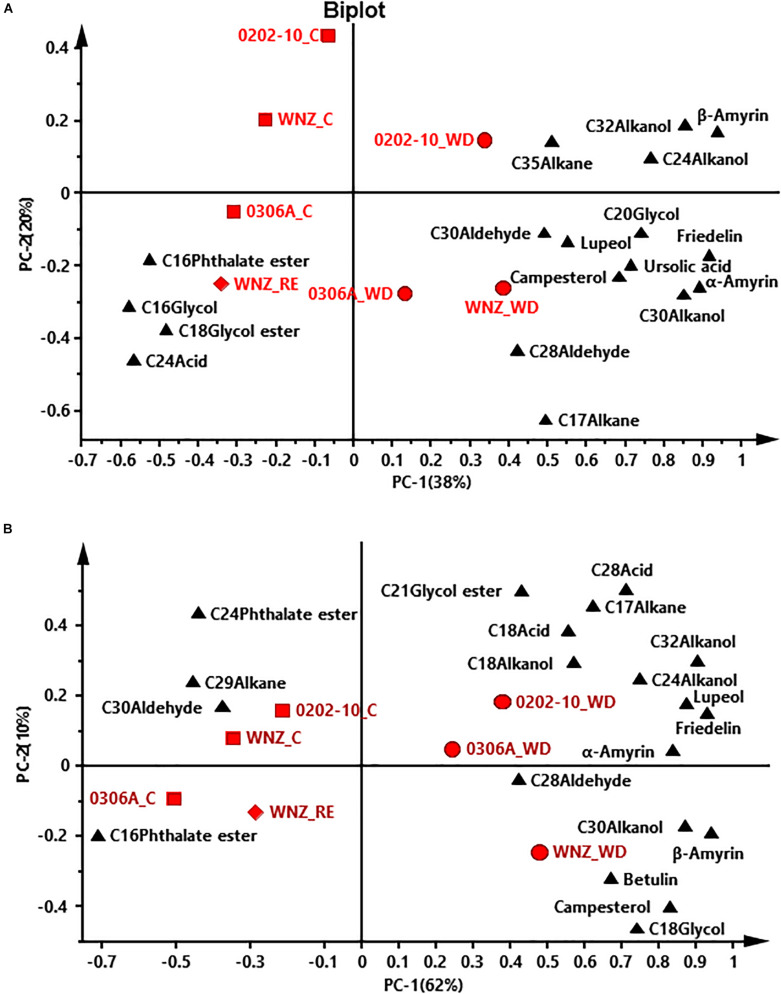
Principal component analysis (PCA) of adaxial intracuticular wax **(A)** and abaxial intracuticular wax **(B)**. WNZ, *Wuniuzao*; C, control, represented by solid square; WD, water deprivation treatment, represented by solid circle; RE, rehydration treatment, represented by solid diamond. The solid triangle represents individual wax position on PCA plot.

To offer an overview of the distinctions among WW control, WD-, and RE-treated plants, the cuticular wax compositions were subjected to principal component analysis (PCA); four PCA models were successfully established for total cuticular waxes, adaxial EWs, adaxial IWs, and abaxial IWs (Figures 6, 7). However, no PCA model could be established for abaxial EWs.

In PCA score plots of total cuticular waxes, adaxial IWs, and abaxial IWs, the first components (PC-1) were able to explain 48%, 38%, and 62% of the variance, respectively; the second principal (PC-2) explained 21%, 20%, and 10% of the variance, respectively (Figures 6A, 7A,B). The WD-treated plants were located on the right side of the plot, primarily due to certain aliphatic compounds and pentacyclic compounds. On the other hand, WW control and rehydration treatment plants were located on the left side of the plot, with sample clustering largely dependent on PC-1 (Figures 6A, 7A,B). PC-1 was primarily driven to the positive direction by triterpenoids, steroids, and certain aliphatic compounds (Figures 6A, 7A,B).

In PCA score plots of adaxial EW, PC-1 and PC-2 expressed 48% and 14% of the variance, respectively. WD-treated *0306A* and *0202-10* were located on the right side of the plot while WD- and RE-treated *Wuniuzao* were located on the left side of the plot together with WW control (Figure 6B). PC-1 was primarily driven to the positive direction by aliphatic compounds, esters, and glycols (Figure 6B).

### Correlation Analysis Between Cuticular Transpiration Rates and Cuticular Waxes

The correlations between cuticular transpiration rates and wax chemical classes from individual cuticular compartments were analyzed. No significant correlations were found from abaxial EWs (Table 1). Five chemical classes, including aldehydes from adaxial EWs and IWs, 1-alkanols from abaxial IWs, 1-alkanol esters from adaxial EWs, triterpenoids from adaxial and abaxial IWs, and steroids from abaxial IWs, were negatively and significantly correlated with cuticular transpiration rates (Table 1). Total wax coverage from adaxial IWs and abaxial IWs also showed significant negative correlations with cuticular transpiration rates, while no correlations were found from total adaxial EWs and abaxial EWs (Table 1).

**TABLE 1:** Correlations (Pearson’s *R*^2^ values) between cuticular transpiration rates and cuticular waxes chemical classes.

	Adaxial surface		Abaxial surface	
	EW	IW	EW	IW
Acids	−0.51	−0.25	−0.07	−0.54
Aldehydes	−0.68*	−0.59*	−0.14	+ 0.03
1-Alkanols	−0.39	−0.32	−0.07	−0.93*
Alkanes	−0.13	+ 0.06	−0.01	+ 0.01
1-Alkanol esters	−0.59*	+ 0.09	−0.08	+ 0.08
Glycol esters	−0.52	−0.00	−0.33	−0.12
Phthalate esters	−0.39	+ 0.15	−0.39	+ 0.26
Glycols	−0.00	+ 0.42	−0.01	−0.03
β-Tocopherol	−0.37	−0.04	−0.07	−0.01
Triterpenoids	−0.53	−0.71*	+ 0.01	−0.88*
Steroids	−0.00	−0.07	+ 0.07	−0.74*
Caffeine	−0.50	+ 0.00	−0.36	+ 0.00
Coverage	−0.51	−0.78*	−0.11	−0.97*

Plus (+) and minus (−) represent positive and negative correlation, respectively. Values labeled with asterisk (*) are significant at P < 0.05. EW, epicuticular waxes; IW, intracuticular waxes; ND, not detected.

The correlations of cuticular transpiration rates with individual wax components from each cuticular compartment were also analyzed. No wax component from abaxial EWs was found to be correlated with cuticular transpiration rates (Table 2). In contrast, 7, 6, and 12 wax components from adaxial EWs, adaxial IWs, and abaxial IWs were found to be significantly correlated with cuticular transpiration rates. These seven wax components from the adaxial EWs included acids (C16, C18), aldehydes (C28, C30), 1-alkanol ester (C22), glycol ester (C21), and glycol (C18); the six wax components from the adaxial IWs included aldehyde (C28), 1-alkanols (C24, C30, C32), phthalate ester (C16), and β-amyrin; the 12 wax components from the abaxial IWs included acids (C18, C28), aldehyde (C28), alkanols (C18, C24, C30, C32), alkane (C17), phthalate ester (C16), and triterpenoids (α-amyrin, β-amyrin, lupeol). In total, 16 wax components were found to be negatively and significantly correlated with cuticular transpiration rates, including three acids (C16, C18, and C28), two aldehydes (C28 and C30), four alkanols (C18, C24, C30, and C32), one alkane (C17), one 1-alkanol ester (C22), one glycol ester (C21), one glycol (C18), and three triterpenoids (α-amyrin, β-amyrin, and lupeol). In contrast, only one wax component (C16 phthalate ester) from both adaxial IWs and abaxial IWs was found to be positively and significantly correlated with cuticular transpiration rates (Table 2). A negative relationship between water loss and 1-alkanol compounds was reported in barley, maize, and alfalfa (Zhang et al., 2005; Hasanuzzaman et al., 2017; Li et al., 2019); the lack of 1-alkanols in *Medicago truncatula* decrease the hydrophobicity of the leaf cuticle (Uppalapati et al., 2012). Thus, 1-alkanols play similar roles for cuticular barrier formation in diverse plant species.

**TABLE 2:** Correlations (Pearson’s *R*^2^ values) between cuticular transpiration rates and different cuticular wax compounds.

	Adaxial surface		Abaxial surface	
	EW	IW	EW	IW
C16 acid	−0.58*	−0.25	−0.25	−0.31
C18 acid	−0.64*	−0.22	−0.01	−0.58*
C28 acid	−0.21	−0.00	−0.24	−0.74*
C28 aldehyde	−0.73*	−0.63*	−0.41	−0.81*
C30 aldehyde	−0.66*	−0.55	−0.01	+ 0.09
C18 alkanol	−0.54	−0.02	−0.15	−0.73*
C24 alkanol	−0.08	−0.79*	ND	−0.70*
C30 alkanol	−0.37	−0.73*	−0.09	−0.72*
C32 alkanol	−0.56	−0.85*	−0.43	−0.90*
C17 alkane	−0.55	−0.53	−0.31	−0.78*
C22 1-alkanol ester	−0.59*	+ 0.09	−0.08	+ 0.08
C21 glycol ester	−0.59*	−0.06	−0.17	−0.51
C16 phthalate ester	−0.40	+ 0.62*	−0.36	+ 0.76*
C18 glycol	−0.74*	−0.09	−0.11	−0.51
α-Amyrin	−0.09	−0.57	+ 0.28	−0.87*
β-Amyrin	−0.52	−0.59*	+ 0.00	−0.75*
Lupeol	ND	−0.57	ND	−0.77*

Plus (+) and minus (−) represent positive and negative correlation, respectively. Values labeled with asterisk (*) are significant at P < 0.05. EW, epicuticular wax; IW, intracuticular wax; ND, not detected.

## Discussion

### Leaf Cuticular Transpiration Barriers Were Reversibly Modified by Multiple Modes in Response to Water Status of Plants

The cuticle is generally perceived as a non-living structure; it remains unclear whether its transpiration barrier properties could be reversibly modified, especially from perennial evergreen plants which often experience annual wet–dry cycles. Extensive wax turnover from adaxial EWs has been reported in *Prunus laurocerasus* during leaf organogenesis (Jetter and Schäffer, 2001). After leaf development completion, the absolute amounts and the relative composition of surface compounds are invariable (Jetter et al., 2000). A recent study showed that drought stress modified tea cuticular waxes of the tender and mature tea leaf through some common and unique modes for transpiration barrier reinforcement (Chen et al., 2020). Here, we found that the leaf cuticular transpiration barrier was reversibly modified in *Wuniuzao* during the WD–RE cycle (Figure 4). Transpiration measurement indicated that after WD treatment, the barrier properties of adaxial EW were improved (Figure 5A). However, its EW coverages did not share a similar tendency as *0202-10* and *0306A* after WD treatment (Figure 1B). Wax analysis demonstrated that although the total adaxial EW coverage from *Wuniuzao* was unaltered by WD, several chemical classes including alkanes, phthalate ester, β-tocopherol, steroids, and caffeine still showed significant changes after WD treatment (Figure 2A, upper panel). The abaxial EW coverage of *Wuniuzao* also showed a different tendency as *0202-10* and *0306A* by WD treatment (Figure 1B); these differences did not alter their transpiration barrier (Figure 5B). Thus, the transpiration barrier modifications of adaxial EW are different from that of abaxial EW. By now, it remains unclear whether the EW barrier adjustment of *Wuniuzao* is a germplasm-specific exception or a general phenomenon, and further characterization of tea germplasm in future studies will required for addressing this question.

The wax coverage from each cuticular compartment showed four general changing patterns: (1) some wax components remained constant regardless of water status changes; (2) wax components were unaffected by WD, but significantly altered by RE treatment; (3) wax components were significantly altered by WD, but unaffected by RE treatment; (4) wax components were reversibly altered by WD and RE (Figures 2A,B, upper panel, 3A,B, upper panel). Next, those wax compounds showing reversible changes from both EWs and IWs of the same leaf side during the WD–RE cycle were examined; alkanes and phthalate ester from adaxial surface and 1-alkanol and phthalate ester from abaxial surface were identified with reversible change between EWs and IWs (Figures 2A,B, 3A,B, upper panel). The alkane coverage of adaxial EWs and adaxial IWs were decreased by WD and increased by RE simultaneously (Figures 2A, 3A, upper panel), suggesting that the reduction of alkanes from adaxial EWs during WD less likely resulted from its transport back into IWs. Otherwise, one would expect the alkane coverage from adaxial IWs to be increased rather than decreased by the WD treatment. The alkanes from adaxial IWs likely have two routes of loss during WD treatment: (1) phase separation into adaxial EWs followed by loss in the surrounding environment; (2) transport back into the epidermal cell wall or protoplast for further transformation. On the other hand, during WD, alkane synthesis or transport from adaxial epidermal cells could be suppressed; this would also contribute to its reduction in adaxial EWs and IWs. After the RE treatment, alkane synthesis or transport from adaxial epidermal cells could be activated, thus leading to simultaneous increases in adaxial EWs and IWs (Figures 2A,3A, upper panel).

The increased coverage of phthalate ester from EWs after WD treatment was accompanied by its simultaneous reduction from IWs (Figures 2A,B, 3A,B); after RE treatment, its EW coverage decrease was accompanied by its IW coverage increase from both leaf surfaces (Figures 2, 3). In contrast, 1-alkanol from the abaxial surface showed opposite trends as phthalate ester: WD treatment decreased its EW coverage while increasing its IW coverage; RE treatment increased its EW coverage while decreasing its IW coverage (Figures 2B, 3B, upper panel). These observations suggest that phthalate ester and 1-alkanol could be phase separated between EWs and IWs in response to water status changes. Because this type of cuticle modification does not require new wax biosynthesis or transport, it is quick while being more carbon and energy efficient.

Triterpenoids from IWs of both leaf surfaces also showed robust reversible changes during the WD–RE cycle in *Wuniuzao*, which was increased by WD and decreased by RE (Figures 3A,B, upper panel). However, the triterpenoids from respective EWs did not show clear correlated changes (Figures 2A,B, upper panel), suggesting that the triterpenoids from IWs cannot be phase separated between EWs and IWs. One possibility is that the synthesis and transport of triterpenoids were activated by WD which led to its large increase in IWs. After RE treatment, IWs triterpenoids could be decreased through three non-exclusive pathways: (1) transport back to the epidermal cell wall or the protoplast, where they are further metabolized or transformed (Jetter and Schäffer, 2001); (2) react with functional groups of cuticle constituents or bind to them hence becoming insoluble (Basford, 1991); (3) due to RE, changes may occur in the cuticle interior (e.g., further cutin polymerization) which reduce triterpenoid extraction by solvents. Under the last scenario, the apparent reduction of triterpenoids from IWs after RE treatment reflects the reduced solvent extraction efficiency that resulted from the changing intracuticular environment (Schmidt and Schönherr, 1982; Kolattukudy, 1984; Khan and Marron, 1988; Riederer and Schönherr, 1988). Meanwhile, triterpenoid synthesis or transport from epidermal cells could be suppressed by RE treatment. To support this notion, steroids from IWs, which derives from same MVA pathway as triterpenoids, was also reduced by RE treatment.

### The Weak Cuticular Transpiration Barriers Were Preferentially Modified in Response to Water Deprivation and Rehydration

In this study, we found that within the same plant species such as *Camellia sinensis* (L.) O. Ktze, each germplasm showed diverse patterns of cuticular wax modification; within the same germplasm, EWs and IWs from adaxial and abaxial sides were also differentially modified in response to WD (Figures 1–3). Notably, WD-induced adaxial EW coverage increases in *0306A* correlated with its transpiration barrier enhancement while no such correlations were observed in *Wuniuzao* or *0202-10* (Figures 1B, upper left, 5A). WD-induced abaxial EW coverage changes did not correlate with their transpiration changes in all the three germplasms (Figures 1B, upper right, 5B). At a wax chemical class level, aldehydes and 1-alkanol esters from adaxial EWs were found to be negatively and significantly correlated with cuticular transpiration rates, while no wax chemical classes from abaxial EWs were found to have such correlation (Table 1). After WD treatment, multiple wax components from adaxial EWs and abaxial IWs were significantly altered and correlated with cuticular transpiration changes (Tables 1, 2). Considering that the cuticular transpiration barriers are located on adaxial IWs and abaxial EWs under normal growth conditions, while adaxial EWs and abaxial IWs contribute to a lower extent to the cuticular transpiration barrier (Vogg et al., 2004; Buschhaus et al., 2015; Jetter and Riederer, 2016; Zhang et al., 2020), our data suggest that the initial weak transpiration barriers were preferentially modified for cuticular transpiration barrier enhancement, an effect known as “make up for shortcomings.” In contrast, the major transpiration barriers (adaxial IWs and abaxial EWs) were either strengthened or unaltered by WD (Tables 1, 2). This cuticular modification mode could have physiological relevance considering that the available carbon and energy resources become limited under drought stress; there would be a tradeoff between carbon and energy cost and the reinforcement of cuticular transpiration barrier.

At the individual chemical level, 7, 6, and 12 wax components from the adaxial EWs, adaxial IWs, and abaxial IWs showed significant correlations with cuticular transpiration rates (Table 2). This suggests that the cuticular transpiration barrier modifications do not require the overhaul of all wax components. Instead, adjusting a small set of specific wax components from individual cuticular compartments is sufficient to alter cuticular transpiration barrier. Interestingly, most wax components showed a negative correlation except C16 phthalate ester, which showed a positive correlation with cuticular transpiration rates (Table 2), suggesting that cuticular transpiration barrier modifications can be realized by either selective deposition or removal of a small set of specific wax components from individual cuticle compartments.

### The Roles of Alicyclic Wax Compounds for Cuticular Transpiration Barrier Modifications

The major cuticular transpiration barrier is formed by very long-chain fatty acid derivative compounds (Vogg et al., 2004; Jetter and Riederer, 2016), which were thought to be mainly located in the tight and highly ordered crystalline zone to make water difficult to diffuse (Riederer and Schneider, 1990). Alicyclic compounds (triterpenoids, steroids, and tocopherols) are mainly present as IWs and positively correlated with cuticular transpiration rates (Vogg et al., 2004; Buschhaus and Jetter, 2012; Jetter and Riederer, 2016). Thus, they were not regarded as contributors to the cuticular transpiration barrier (Riederer and Schneider, 1990; Jetter and Riederer, 2016). In this study, we found that triterpenoids and steroids from IWs were negatively and significantly correlated with cuticular transpiration rates (Tables 1, 2), which is in accordance with a recent report showing that cuticular transpiration is correlated with triterpenoids in the IWs rather than the EWs (Schuster et al., 2016; Zeisler and Schreiber, 2016; Romero and Rose, 2019). Under our experimental conditions, the tea plants were subjected to multiple stresses including dehydration, high temperature, and high light radiation (Supplementary Figures 1A, 2). The increases in temperature will decrease the strength and rigidity of the cuticle (Edelmann et al., 2005). In addition, the loss of leaf turgor pressure generates considerable mechanical stress to the cuticle. It is critical to maintain cuticle structural intact to avoid barrier failure. This raises an interesting question: how does the WD-induced alicyclic compound deposition in IWs confer improved transpiration barrier together with mechanical strength? One possibility is that triterpenoids could serve as fillers restricting the mobility of cutin and polysaccharide chains (Tsubaki et al., 2013); this would increase cuticle stiffness and breaking stress, and decrease its maximum strain. Another possibility is that drought stress drives triterpenoid polymerization with cutin, thus resulting in the depolymerization of cutin polymer itself; this would increase cuticle stiffness and breaking stress in a similar mechanism as tomato cuticle during fruit ripening (España et al., 2014). Because pectin has been demonstrated to present in the cuticles of leaves of some species and tomato fruit, the acid groups of this polysaccharide may also facilitate chemical binding at least for cutin, waxes, or phenols, and the chemical scenario could be more complex than what we speculate previously. The polysaccharides of CEPs have been demonstrated to be responsible for cuticle water permeability (Domínguez and Heredia, 1999) due to a net negative charge conferred to the cuticle which influences water sorption and cuticular transport (Heredia and Benavente, 1991). In tomato cuticle, the hydroxyl functional group of flavonoids plays an important role in the control of water transport across the polymer matrix (Luque et al., 1995). As suggested for flavonoids, the existing hydroxyl groups of triterpenoids may contribute to neutralizing cuticle charge and restrict cuticular transpiration. In addition, triterpenoids have a planar conformation which makes them easily embedded into the hydrophobic interplanar space of cutin matrix and restrict water transport across the CEP matrix. We hypothesized that through these different mechanisms, triterpenoids could contribute as cuticle transpiration barrier and simultaneously provide mechanical strength to cope with multiple stresses induced by water deprivation.

Steroid coverage in abaxial IWs was increased by WD (Figure 3); similar observations are also reported in barley leaves under drought and heat stress (Kuczyńska et al., 2019). Plant sterols can increase the rigidity of the cell wall and regulate the fluidity and permeability of cell membranes to limit transpiration under stress conditions (Hartmann, 1998; Hussain et al., 2018). Sterols present in the cuticle could play similar roles as their counterparts in cell walls or lipid bilayers, ultimately limiting cuticular transpiration.

In summary, we showed that tea leaf cuticular transpiration barriers were reversibly modified in response to different water availability regimes. After drought, the weak cuticular transpiration barriers were preferentially reinforced, while the major cuticular transpiration barriers were either strengthened or unaltered. Subsequently, cuticular waxes were plastically modified in response to plant water status via multiple mechanisms, but adjustment of a small set of intracuticular or epicuticular waxes was sufficient to modify the overall leaf cuticular transpiration barrier.

## Data Availability Statement

The original contributions presented in the study are included in the article/Supplementary Material, further inquiries can be directed to the corresponding author.

## Author Contributions

MC and YZ conceived the original research plans, designed the experiments, and analyzed the data. MC, CC, and WS supervised the experiments. YZ, YH, XC, and XK performed the experiments. ZD provided technical assistance to YZ. YZ wrote the article with contributions of all the authors. MC supervised and completed the writing, agrees to serve as the author responsible for contact and ensures communication. All authors contributed to the article and approved the submitted version.

## Conflict of Interest

The authors declare that the research was conducted in the absence of any commercial or financial relationships that could be construed as a potential conflict of interest.

## Supplementary Material

Supplementary Figure 1 The daily temperature **(A)** and humidity **(B)** changes inside the glass house during the experiment.

Supplementary Figure 2 The relative leaf contents and morphological changes of *Wuniuzao*, *0306A*, and *0202-10* during water deprivation and rehydration treatments. **(A)** Absolute soil water content changes during the 12-day water deprivation treatment. **(B)** Relative leaf water contents from *Wuniuzao*, *0306A*, and *0202-10* during water deprivation and rehydration treatments. **(C)** Morphological changes of *Wuniuzao*, *0306A*, and *0202-10* at day 1, day 8, and day 12 of water deprivation as well as at day 10 after rehydration.
