# Supplementary material for: Plasticity of the Cuticular Transpiration Barrier in Response to Water Shortage and Resupply in Camellia sinensis: A Role of Cuticular Waxes
Source: Front Plant Sci. 2021 Jan 11;11:600069. doi: 10.3389/fpls.2020.600069 (PMC7829210; doi:10.3389/fpls.2020.600069)
Supplement: Supplementary Figure 1 — The daily temperature (A) and humidity (B) changes inside the glass house during the experiment. [file Presentation_1.PPTX]

## Slide 1
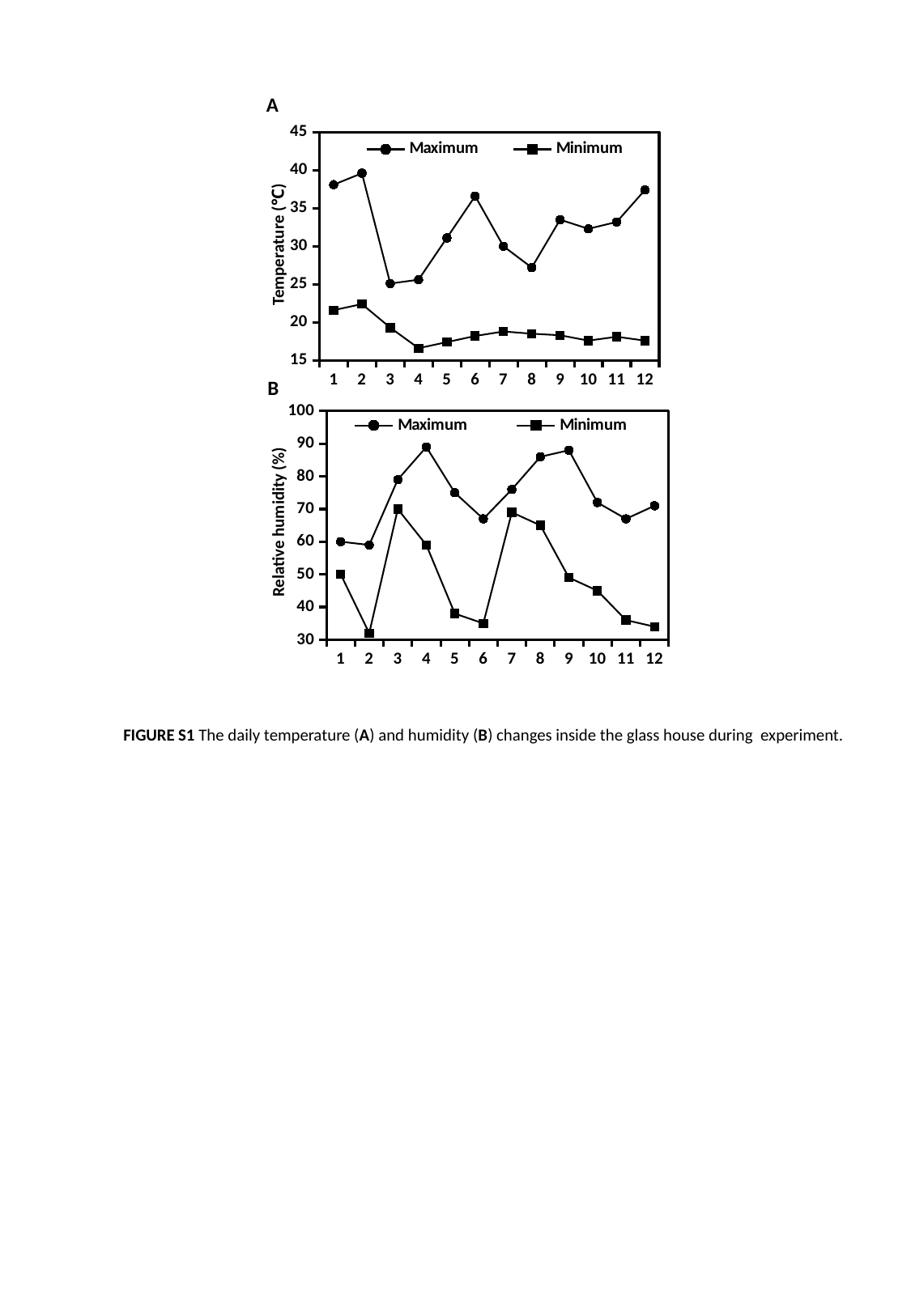

A
### Chart
| Category | Maximum | Minimum |
|---|---|---|
| 1 | 38.1 | 21.6 |
| 2 | 39.6 | 22.4 |
| 3 | 25.1 | 19.3 |
| 4 | 25.6 | 16.6 |
| 5 | 31.1 | 17.4 |
| 6 | 36.6 | 18.2 |
| 7 | 30.0 | 18.8 |
| 8 | 27.2 | 18.5 |
| 9 | 33.5 | 18.3 |
| 10 | 32.3 | 17.6 |
| 11 | 33.2 | 18.1 |
| 12 | 37.4 | 17.6 |Temperature (℃)
B
### Chart
| Category | Maximum | Minimum |
|---|---|---|
| 1 | 60.0 | 50.0 |
| 2 | 59.0 | 32.0 |
| 3 | 79.0 | 70.0 |
| 4 | 89.0 | 59.0 |
| 5 | 75.0 | 38.0 |
| 6 | 67.0 | 35.0 |
| 7 | 76.0 | 69.0 |
| 8 | 86.0 | 65.0 |
| 9 | 88.0 | 49.0 |
| 10 | 72.0 | 45.0 |
| 11 | 67.0 | 36.0 |
| 12 | 71.0 | 34.0 |Relative humidity (%)
Figure S1 The daily temperature (A) and humidity (B) changes inside the glass house during experiment.

## Slide 2
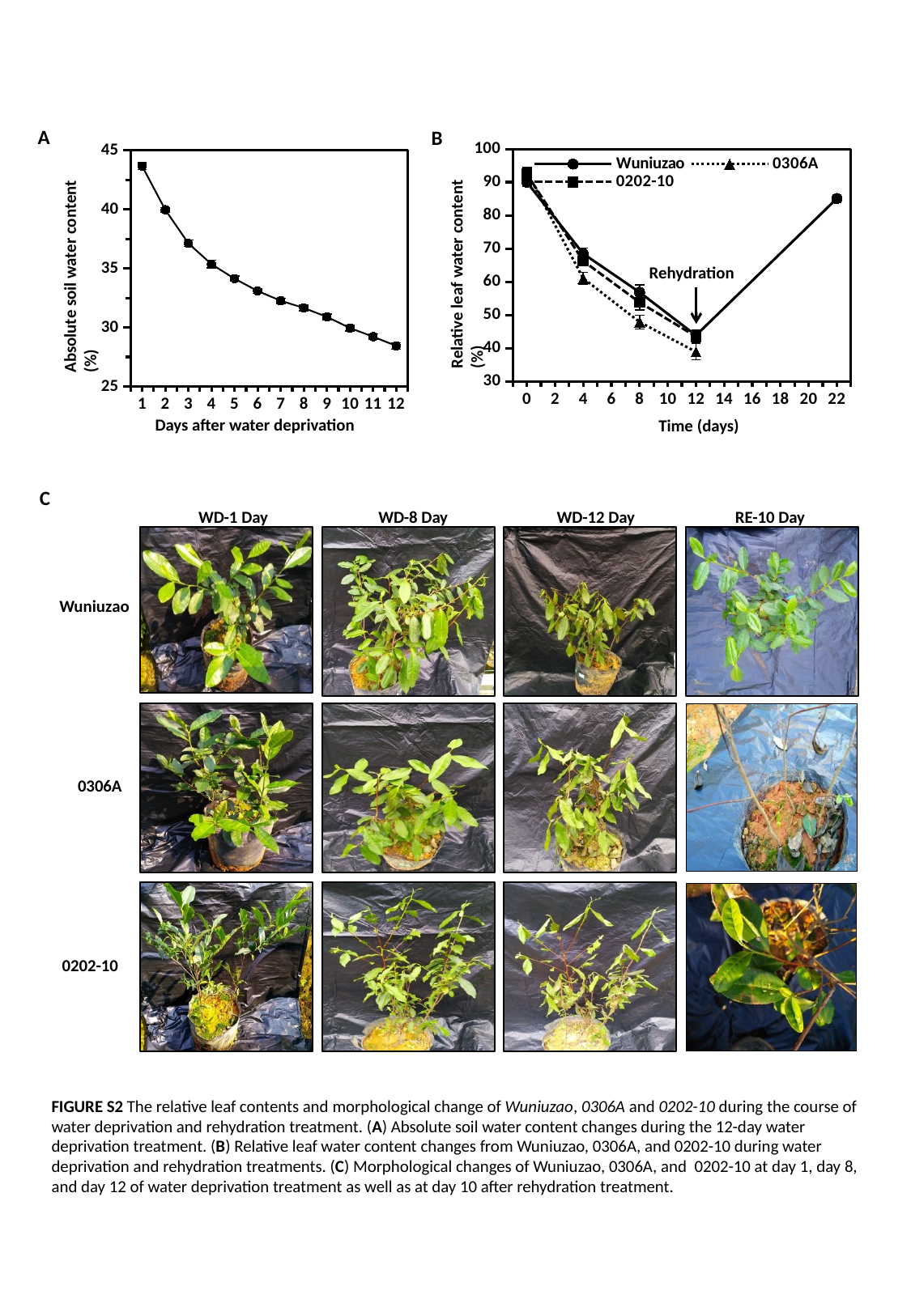

A
B
### Chart
| Category | AVE |
|---|---|
| 1 | 43.66297081218877 |
| 2 | 39.96771168277034 |
| 3 | 37.12619958366844 |
| 4 | 35.360083504011605 |
| 5 | 34.1286647899895 |
| 6 | 33.089044574114546 |
| 7 | 32.25814387365182 |
| 8 | 31.642397199655992 |
| 9 | 30.896718270297267 |
| 10 | 29.94855137427683 |
| 11 | 29.217050949320246 |
| 12 | 28.439539339414686 |Absolute soil water content (%)
Days after water deprivation
### Chart
| Category | Wuniuzao | 0306A | 0202-10 |
|---|---|---|---|
| 0 | 89.9968161984779 | 93.0728123917618 | 92.4255020902336 |
| 2 | None | None | None |
| 4 | 68.4540198811636 | 61.0561864797305 | 66.2801031781998 |
| 6 | None | None | None |
| 8 | 56.826506092418605 | 47.8701793932563 | 53.7882260761335 |
| 10 | None | None | None |
| 12 | 43.8474159909276 | 38.9074803225015 | 43.5236927697949 |
| 14 | None | None | None |
| 16 | None | None | None |
| 18 | None | None | None |
| 20 | None | None | None |
| 22 | 85.0807118787623 | None | None |Relative leaf water content (%)
Rehydration
Time (days)
C
RE-10 Day
WD-12 Day
WD-1 Day
WD-8 Day
Wuniuzao
0306A
0202-10
Figure S2 The relative leaf contents and morphological change of Wuniuzao, 0306A and 0202-10 during the course of water deprivation and rehydration treatment. (A) Absolute soil water content changes during the 12-day water deprivation treatment. (B) Relative leaf water content changes from Wuniuzao, 0306A, and 0202-10 during water deprivation and rehydration treatments. (C) Morphological changes of Wuniuzao, 0306A, and 0202-10 at day 1, day 8, and day 12 of water deprivation treatment as well as at day 10 after rehydration treatment.
